# Is automated platelet counting still a problem in thrombocytopenic blood?

**DOI:** 10.1590/S1516-31802003000100005

**Published:** 2003-01-02

**Authors:** Raimundo Antônio Gomes Oliveira, Maria Mariko Takadachi, Kimiyo Nonoyama, Orlando César de Oliveira Barretto

**Keywords:** Platelet counting, Automatic counters, Transfusion, Thrombocytopenic patients, Contagem, Plaquetas, Automação, Transfusão, Plaquetopenia

## Abstract

**CONTEXT:**

Reliable platelet counting is crucial for indicating prophylactic platelet transfusion in thrombocytopenic patients.

**OBJECTIVE:**

To evaluate the precision and accuracy of platelet counting for thrombocytopenic patients, using four different automated counters in comparison with the Brecher & Cronkite reference method recommended by the International Committee for Standardization in Hematology (ICSH).

**TYPE OF STUDY:**

Automated platelet counting assessment in thrombocytopenic patients.

**SETTING:**

Hematology Laboratory, Hospital do Servidor Público Estadual de São Paulo, and the Hematology Division of Instituto Adolfo Lutz, São Paulo, SP, Brazil.

**MAIN MEASUREMENTS:**

Brecher & Cronkite reference method and four different automated platelet counters.

**PARTICIPANTS:**

43 thrombocytopenic patients with platelet counts of less than 30,000/μl

**RESULTS:**

The ADVIA-120 (Bayer), Coulter STKS, H1 System (Technicom-Bayer) and Coulter T-890 automatic instruments presented great precision and accuracy in relation to laboratory thrombocytopenic samples obtained by diluting blood from normal donors. However, when thrombocytopenic patients were investigated, all the counters except ADVIA (which is based on volume and refraction index) showed low accuracy when compared to the Brecher & Cronkite reference method (ICSH). The ADVIA counter showed high correlation (r = 0.947). However, all counters showed flags in thrombocytopenic samples.

**CONCLUSION:**

The Brecher & Cronkite reference method should always be indicated in thrombocytopenic patients for platelet counts below 30,000 plt / μl obtained in one dimensional counters.

## RESUMO

**CONTEXTO:**

A contagem de plaquetas confiável é de grande importância para avaliar a necessidade da transfusão profilática em pacientes plaquetopênicos.

**OBJETIVO:**

Avaliar, em amostras plaquetopênicas, a precisão e exatidão da contagem de plaquetas em quatro contadores automáticos em comparação com método de referência de Brecher & Cronkite recomendado pelo Comitê Internacional de Estandardização em Hematologia.

**TIPO DE ESTUDO:**

Avaliação da contagem automatizada de plaquetas em pacientes trombocitopênicos.

**LOCAL:**

Hospital do Servidor Público Estadual (São Paulo - SP). Instituto Adolfo Lutz.

**PARTICIPANTES:**

43 pacientes trombocitopênicos com contagens de plaquetas inferiores a 30.000/μL.

**VARIAVEIS ESTUDADAS:**

Método de Brecher & Cronkite como padrão de referência e quatro contadores automáticos.

**RESULTADOS:**

Os contadores automáticos ADVIA-120 (Bayer), Coulter STKS, H1 System (Technicom-Bayer) e Coulter T-890 demonstraram boa precisão e exatidão em amostras plaquetopênicas obtidas em laboratório de hematologia a partir de amostras normais. Apenas o ADVIA-120, que utiliza dois princípios de contagem (volume e ín-dice de refração), demonstrou boa correlação com o método de referência recomendado pelo Comitê Internacional de Estandardização em Hematologia (ICSH, 1984/1988) para as amostras dos pacientes trombocitopênicos (r = 0,947). Entretanto, todos os aparelhos pediram nova contagem de plaquetas (flags) para as amostras trombocitopênicas.

**CONCLUSÃO:**

A utilização do método de referência de Brecher Cronkite deve ser uma conduta rotineira e indispensável em todos pacientes trombocitopênicos com contagens abaixo de 30,000 plaq /μl obtidas em contadores que utilizam-se apenas do volume como princípio de contagem.

## INTRODUCTION

It is widely accepted that automation has afforded high precision and accuracy for platelet counting in normal individuals.^1-5^ How-ever, automated counting is still very controversial in the case of samples from thrombocytopenic or other patients in which other small particles could generate electrical or optical signals that are similar to platelets, such as debris and red cell fragments.^4,6-11^ Most counters nowadays employ the principle of electrical impedance or optical signals for counting the platelets in peripheral blood, using the particle volume for counting them.^12^ On the other hand, the presence of large platelets beyond the upper threshold may lead to underestimation of the platelet counts.^13-15^ The use of multiple light scatter parameters rather than impedance alone has improved the ability to discriminate platelets.^6^

Prophylactic platelet transfusions have been successfully employed in hematological patients under chemotherapy when the platelet levels drop to lower than 20,000/μL. Nevertheless, in an attempt to lower the risks in platelet transfusions in bone marrow transplants, as well as reducing the cost, there is a great tendency to use 10,000/μL,^16-21^ or even 5,000/μL as advocated by Gmür et al.,^22^ as the threshold for prophylactic or therapeutic platelet transfusions. Thus, higher precision and accuracy in platelet counting is required.^6^ In fact, the Consensus Conference on Platelet Transfusion Therapy of the National Institute of Health,^23^ reported that there was a lack of reproducibility and a variability in platelet counts at low levels. This fact is a great problem in recommending a standard threshold for platelet transfusion in thrombocytopenic patients.

Manual platelet counting in the Neubauer chamber, by means of a phase-contrast microscope,^24,25^ has been recommended as the reference method for assessing the platelet number by the International Committee for Standardization in Hematology (ICSH -1984).^26^ Quite recently, the International Council for Standardization in Hematology and the International Society for Laboratory Hematology^27^ have recommended the use of labeled platelets in a fluorescence-flow cytometer, together with a semiautomated, single-channel aperture-impedance counter as the reference method for platelet counting, but few centers are able to afford this.

This investigation was thus carried out with the objective of studying the accuracy and precision of automated instruments and comparing these with the recommended manual method (ICSH 1984) for low platelet counts. Different instruments based on different technical characteristics, such as refraction index and platelet size, were used.

## METHODS

Two different materials were employed:

1. Blood samples from four normal individuals were diluted with isotonic solution in order to make target low- platelet suspensions (30,000; 20,000; 10,000 and 5,000 platelets per μL), in accordance with Lawrence et al.^16^ Every target sample was counted 9 times (3 dilutions in triplicate).
2. Blood samples from 43 thrombocyto-penic patients presenting less than 30,000 platelets per μL, 33 of them presenting leukemia and 10 with several diseases such as idiopathic thrombocytopenic purpura, myelodysplastic syndrome and pancytopenia, from the Hematology Laboratory of Hospital do Servidor Público Estadual de São Paulo (HSPE), São Paulo, were also studied.

Four automated hematology analyzers were studied: ADVIA^TM^ 120 Hematology System (Bayer, Tarrytown, New York, USA),^10,15^ H1 Technicon System (Technicon Instrument Corporation/ Tarrytown, New York),^28-30^ Coulter STKS (Coulter, USA),^31^ and Coulter T-890 (Coulter, USA),^32^ as well as the reference method recommended by the International Committee for Standardization in Hematology (1984): the Brecher & Cronkite method.^24,25^

The precision and accuracy of all blood cell counters were assessed daily in comparison with standards provided by the manufacturers. All blood samples from thrombocytopenic patients were processed within 1 hour after blood draw for automated methods, and up to 3 hours for the manual counts, at room temperature. All counts were performed in triplicate. For the reference method (ICSH 1984), a minimum of 200 cells was counted in the Neubauer chamber.

Every instrument was compared with the reference method by a linear correlation test. The Student "t" test was employed for comparisons between all instrument data and for data obtained using the reference method as well, with a significance level of 5%.

## RESULTS

### Laboratory thrombocytopenic samples from normal donors

The ADVIA, STKS and H1 counters showed variable differences between the obtained mean values and the target values, ranging from 1.4% to 5.4% for the 5,000 target group, from −2.3% to 0.3% for the 10,000 target group, from −4.5% to 1.2% for the 20,000 target group, and from −1.6% to −0.1% for the 30,000 platelets per μL target group. The T-890 counter, however, showed mean values from 11 to 16.5% lower than the target values, for the 10,000 to 30,000 platelets per μL target groups. For the 5,000-target group, the results were 19.05% lower than the target value (Tables 1 and 2).

**Table 1:** Platelet counts using the ADVIA-120, STKS, H1 and T-890 systems, in target thrombocytopenic blood samples obtained in the Hematology Laboratory of Hospital do Servidor Público Estadual de São Paulo.

Target Counter values		Platelet * # (x 1000/µL)	**CV% ± SD	K	Platelet Correction ## (x 1000/µL)	Platelet Range (x 1000/µL)
5,000	ADVIA	5.4 ± 0.3	7.0 ± 5.4	0.98	5.18	4 – 7
	STKS	5.2 ± 0.7	7.1 ± 3.4	0.98	5.27	4 – 7
	H1	4.9 ± 0.2	9.3 ± 5.0	0.97	5.07	4 – 6
	T-890	4.1 ± 0.2	7.5 ± 4.2	0.99	4.20	3 – 5
10,000	ADVIA	10.6 ± 0.3	6.5 ± 3.5	0.97	10.01	8 - 12
	STKS	9.5 ± 0.8	4.7 ± 2.1	0.97	9.77	8 - 12
	H1	9.8 ± 0.6	7.7 ± 3.0	0.98	10.03	8 - 12
	T-890	8.4 ± 0.3	2.8 ± 2.3	1.01	8.36	8 - 10
20,000	ADVIA	21.0 ± 1.1	5.7 ± 0.7	0.97	20.24	18- 25
	STKS	18.6 ± 0.9	2.3 ± 0,5	0.97	19.13	17- 21
	H1	19.2 ± 0.4	4.7 ± 1.2	1.01	19.10	18- 22
	T-890	17.1 ± 0.5	3.8 ± 0.5	1.00	17.11	15-19
30,000	ADVIA	30.8 ± 1.3	3.3 ± 0.7	0.98	29.97	26- 35
	STKS	28.9 ± 0,8	3.6 ± 1,0	0.98	29.54	25- 31
	H1	30.1 ± 1.1	4.5 ± 0.9	1.02	29.52	27- 36
	T-890	26.8 ± 0.8	2.1 ± 0.8	1.01	26.69	24- 31

*K (dilution control constant): K > 1 artefactual concentration; K < 1 artefactual dilution;*

* *9 counts for each sample (3 dilutions, each one in triplicate); n = 4 representing serial dilutions of blood samples from 4 different donors.*

# *mean ± SD;*

## *after correction by "K";*

** *Mean of CVs (coefficient of variation) of four samples in each target group.*

**Table 2:** Percentile difference between target and obtained values of platelet counts, for all counters in the target thrombocytopenic blood sample groups, obtained in the Hematology Laboratory of Hospital do Servidor Público Estadual de São Paulo.

Counters	Target groups (platelet/µl)			
	5,000	10,000	20,000	30,000
**Percentile difference (%)**				
ADVIA	+3.6	+0.1	+1.2	−0.1
STKS	+5.4	−2.3	−4.35	−1.53
H1	+14	+0.3	−4.5	−1.6
T-890	−19.05	−16.4	−14.5	−11.03

The coefficients of variations shown by the groups, for all the counters, were lower than 9.5% for the 5,000-target group, lower than 7.8% for the 10,000-target group, lower than 5.8% for the 20,000-target group and lower than 4.6% for the 30,000 platelets per ml target group (Table 1).

The dilutions of the platelet suspensions were checked by the linear correlation test and showed values of r > 0.99 for all counters. The "y" axis intercepts, which represent the number of platelets per μl, were close to zero for all counters. The slope was close to 1, except for T-890 (slope = 0.88).

### Samples from thrombocytopenic patients

The mean value of the platelet counts performed in triplicate by the Brecher & Cronkite reference method was 18,040 platelets per μl. As can be observed in Table 3, ADVIA and STKS showed little deviation, but H1 and T-890 exhibited greater deviation.

**Table 3:** Accuracy analysis: paired "t" test for thrombocytopenic patients with less than 30,000 platelets/µl.

Methods		Mean difference	95%	Confidencet	
A	B	(A – B) platelets/µl	Interval, platelets/µl	(A x B)	p
ICSH	ADVIA	730	−320 to 1,770	1.4	0.168
ICSH	STKS	−940	−2,700 to 810	−1.1	0.286
ICSH	H1	−5,160	−6,720 to –3,610	−6.7	<0.001
ICSH	T890	−6,120	−9,490 to −2,740	−3.7	<0.001

*(A) = 18,040 platelets/ml obtained using the ICSH (Brecher-Cronkite) reference method.; ICSH = International Committee for Standardization in Hematology.*

**Figure 1:**
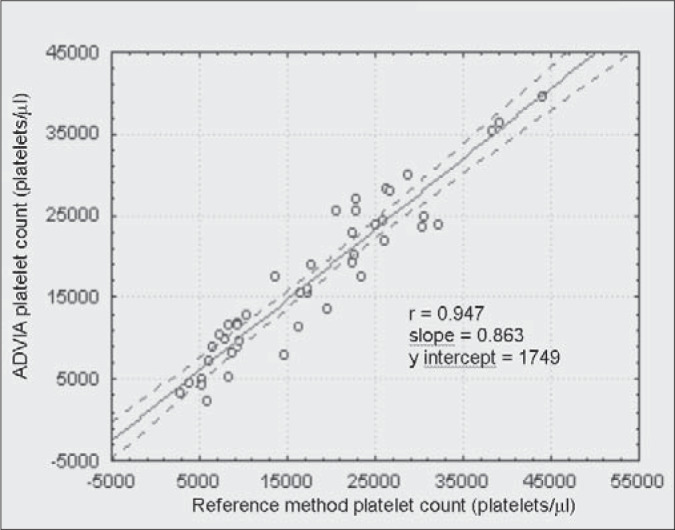
Comparison between the ADVIA counter and the International Committee for Standardization in Hematology reference method in thrombocytopenic patients.

**Figure 2:**
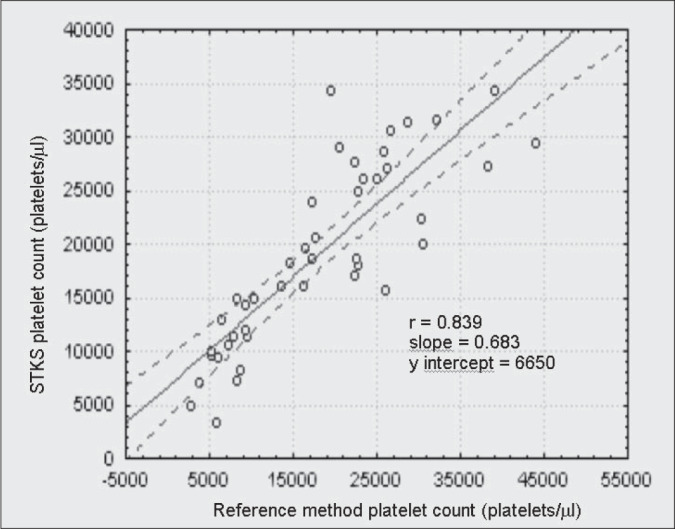
Comparison between the STKS counter and the International Committee for Standardization in Hematology reference method in thrombocytopenic patients.

**Figure 3:**
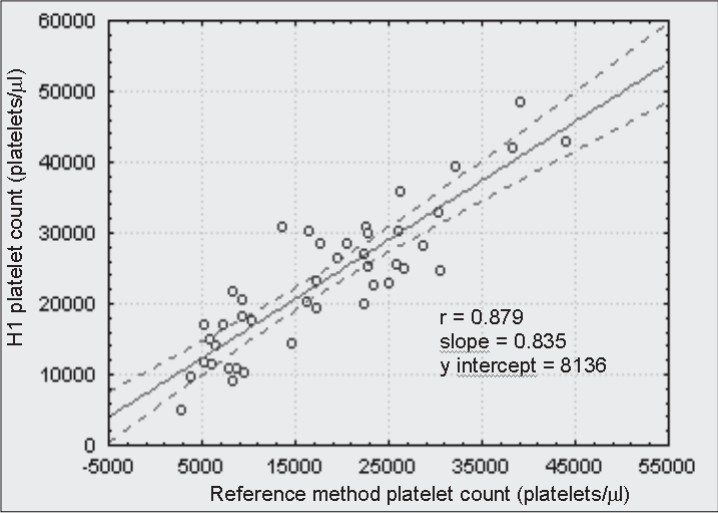
Comparison between the H1 counter and the International Committee for Standardization in Hematology reference method in thrombocytopenic patients.

**Figure 4:**
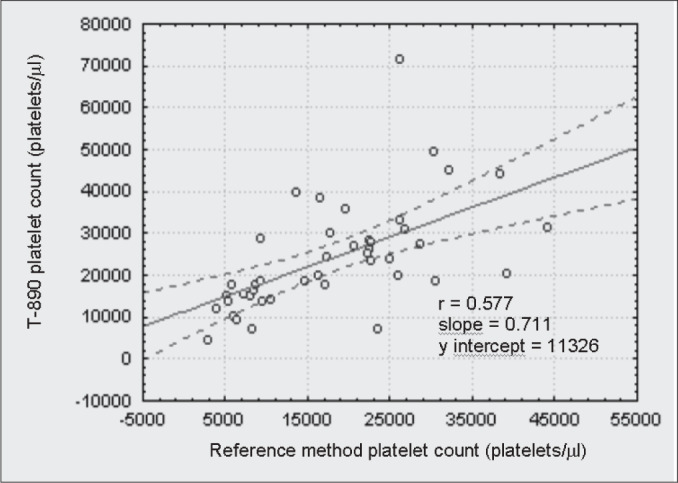
Comparison between the T-890 counter and the International Committee for Standardization in Hematology reference method in thrombocytopenic patients.

The linear correlation test between every counter and the reference method for thrombocytopenic patients are shown in Figures 1, 2, 3 and 4. The ADVIA counter exhibited the highest correlation (r = 0.947).

## DISCUSSION

With regard to the laboratory targets for platelet counting, the different counters used indicated great accuracy and precision. However, the Coulter T-890 exhibited 11 to 19.5% of the data lower than the desired target values (Tables 1 and 2), similar to what was obtained by Lawrence^16^ using a counter that also employed the impedance principle.

When dealing with the thrombocytopenic patient samples, comparative determinations between the automated methods and the reference method suggest that the two-dimensional counting system employed by the ADVIA counter demonstrates higher accuracy in differentiating between platelet and non platelet particles, in comparison with the one-dimensional system used by H1, STKS and T-890 (Figures 1, 2, 3 and 4). The data herein presented are similar to those obtained by Kunicka et al.^10^Dickerhoff and von Ruecker^4^ also showed lower correlation between the H1 counter and flow cytometry (FC) with monoclonal anti-platelet antibodies, when using thrombocytopenic samples of lower than 50,000 platelets per μL. Only FC and the Brecher & Cronkite method showed significant correlation. Interestingly, these are the two reference methods recommended by the ICSH.

Hanseler et al.^11^ using the H1 counter, claimed that for counts of less than 30,000 platelets per μL, the automated counting should be replaced by the manual chamber procedure. Our data obtained with thrombocytopenic patients also suggest the same for the one-dimensional STKS, H1 and T-890 counters.

The data from Ault^6^ and Kunicka et al.,^10^ as well as the data obtained in this investigation for H1, STKS and T-890, suggest that the one-dimensional platelet counters present a tendency to overestimate the platelet counts when other particles with the same platelet size are contaminating the sample. However, all counters showed flags in thrombocytopenic samples.

## CONCLUSION

Our results suggest that for platelet counts below 30,000 platelets per μl obtained in one-dimensional counters, the counting method should be replaced by the reference manual procedure, i.e. the Brecher & Cronkite method.
